# Correlation between Gastric Mucosal Morphologic Patterns and Histopathological Severity of *Helicobacter pylori* Associated Gastritis Using Conventional Narrow Band Imaging Gastroscopy

**DOI:** 10.1155/2015/808505

**Published:** 2015-05-18

**Authors:** Taweesak Tongtawee, Soraya Kaewpitoon, Natthawut Kaewpitoon, Chavaboon Dechsukhum, Ryan A. Loyd, Likit Matrakool

**Affiliations:** ^1^Endoscopic Unit, Department of Surgery, Institute of Medicine, Suranaree University of Technology, Nakhon Ratchasima 30000, Thailand; ^2^Suranaree University of Technology Hospital, Nakhon Ratchasima 30000, Thailand; ^3^Department of Family Medicine and Community Medicine, Suranaree University of Technology, Nakhon Ratchasima 30000, Thailand; ^4^Parasitic Disease Research Unit, Suranaree University of Technology, Nakhon Ratchasima 30000, Thailand; ^5^Faculty of Public Health, Vongchavalitkul University, Nakhon Ratchasima 30000, Thailand; ^6^Department of Pathology, Institute of Medicine, Suranaree University of Technology, Nakhon Ratchasima 30000, Thailand

## Abstract

*Background and Aim*. Identifying specific gastric mucosal morphologic patterns useful for detecting *Helicobacter pylori* associated gastritis and correlation with histopathological severity. *Methods*. The endoscopists classified the C-NBI gastroscopic findings into 5 gastric mucosal morphologic patterns as follows: type 1: regular arrangement of collecting venules, type 2: cone-shaped gastric pits, type 3: rod-shaped gastric pits with prominent sulci, type 4: ground glass-like morphology, and type 5: dark brown patches with bluish margin and irregular border. Biopsies of all of the cases were then evaluated by 5 pathologists for definitive *Helicobacter pylori* diagnosis. *Result*. Type 1 and type 2 patterns were statistically significant in predicting *Helicobacter pylori* negative status (58/60, *P* < 0.01). Type 3, type 4, and type 5 patterns were statistically significant in predicting *Helicobacter pylori* positive status (132/140, *P* < 0.01). Furthermore, the sensitivity, specificity, and positive and negative predictive values of type 3, 4, or 5 morphologies for predicting *Helicobacter pylori* positive were 94.28%, 96.66%, 98.50%, and 87.87%, respectively, correlated well with inflammation grading according to the Sydney classification (*P* < 0.01). *Conclusion*. Our study suggests that gastric mucosal morphologic patterns in the *Helicobacter pylori* infected gastric mucosa can be reliably identified using C-NBI gastroscopy with good correlation with inflammation grading.

## 1. Introduction

Narrow band imaging (NBI) is an optical image enhancement technique that enhances the vessels and patterns of the gastric mucosa surface. Since the discovery of* Helicobacter pylori *in 1983, strong evidence has indicated that* Helicobacter pylori* infection plays an important role in the pathogenesis of chronic gastritis, peptic ulcer disease, and gastric malignancy [1, 2]. European guidelines [3] indicate that at least 2 different tests are necessary to make the diagnosis of* Helicobacter pylori* infection. Although gastroscopic features of* Helicobacter pylori* associated gastritis have been reported in the literature, there is a controversy whether* Helicobacter pylori* associated gastritis can be diagnosed by gastroscopic features alone. Most studies have concluded that it is impossible to diagnose* Helicobacter pylori* related gastritis on the basis of gastroscopic findings alone [4–7]. The narrow band imaging (NBI) system is an endoscopic imaging technique for enhanced visualization of mucosal microscopic structure and capillaries in the superficial mucosal layer. Images are obtained by using narrow band red, blue, and green filters, which are different from conventional red-green-blue filters [8]. Some recent study from the United States has indicated the usefulness of high resolution narrow band imaging gastroscopy for predicting* Helicobacter pylori* infection and the occurrence of intestinal metaplasia in the stomach [9]. However, in daily clinical practice, the high resolution gastroscopy does not seem to be feasible, because it takes more examination time and needs more training and experience of the endoscopist. If specific gastric mucosal morphologic patterns of* Helicobacter pylori* associated gastritis can be identified using C-NBI gastroscopy, this may be useful for “*site specific biopsy*” of areas with suspected* Helicobacter pylori* infection in daily practice. Up to the present time, there has not been strong evidence regarding specific gastric mucosal morphologic patterns of* Helicobacter pylori* associated gastritis using C-NBI gastroscopy. The aim of this study is to identify specific gastric mucosal morphologic pattern for the detection of* Helicobacter pylori* associated gastritis and to evaluate the possible correlation between these gastroscopic findings and histopathological severity using C-NBI gastroscopy as well as the correlation with inflammation grading according to the Sydney classification [10].

## 2. Materials and Method

### 2.1. Patients

A total of 200 patients who underwent gastroscopy for the investigation of dyspeptic symptoms were enrolled in the study from January 2014 to November 2014 at the Endoscopic Unit, Department of Surgery, Suranaree University of Technology Hospital (SUTH), Institute of Medicine, Suranaree University of Technology, Nakhon Ratchasima, Thailand. The following exclusion criteria were applied: age below 18 or above 70 years,* Helicobacter pylori* eradication treatment in the previous 2 months, gastric ulcer or duodenal ulcer, suspected or confirmed malignancy on endoscopy, significant medical illnesses and history of previous gastric surgery, and the use of antimicrobials or gastrointestinal medications like PPIs, H2 blockers, or bismuth compounds within the previous 2 months. All patients provided informed consent, and the study was approved by the Institutional Review Board of Suranaree University of Technology, Nakhon Ratchasima, Thailand. The study was performed in accordance with good clinical practice and the guidelines of the Declaration of Helsinki. All patients provided a written informed consent and the study protocol was approved by the Ethics Committee for Research Involving Human Subjects, Suranaree University of Technology (EC-57-22).

### 2.2. Diagnosis of* Helicobacter pylori* Infection

A diagnosis of* Helicobacter pylori* infection was made if* Helicobacter pylori* bacteria were seen on both histopathological examinations and the rapid urease test was positive. A recent study from India attempted to define the gold standard of diagnostic tests to determine* Helicobacter pylori* infection status by breaking down the respective sensitivities and specificities. Both sensitivity and specificity of nested PCR have been reported to be 100%. In contrast, the sensitivity and specificity of serological, urea breath, fecal antigen, rapid urease tests, histopathology, PCR, and culture have been found to be 85% and 79%, 75%–100% and 77%–100%, 67%–100% and 61%–100%, 75%–100% and 84%–100%, 66%–100% and 94%–100%, 75%–100% and 84%–100%, and 55%-56% and 100%, respectively [10]. PCR does not seem to be feasible in daily clinical practice; thus we chose in our study patients to consider them* Helicobacter pylori* negative if they had negative results in one or both of the above selected tests.

### 2.3. Biopsy Specimens

Four biopsy samples were taken directly from the observation sites as shown in Figures 3 [Fig fig4] [Fig fig5] [Fig fig6]–7. Two samples were sent for histological analysis and 2 were used for rapid urease testing on site (Prontodyle, GASTREX, France).

### 2.4. Histological Analysis

Specimens for histological analysis were placed in 10% formalin solution and routinely processed. The hematoxylin and eosin stain and Giemsa stain were used for identification of* Helicobacter pylori*. All of the cases were evaluated by 5 pathologists from Bangkok Pathological Laboratory outside Suranaree University according to the Sydney classification (Table 1), including evaluation of chronic inflammation, atrophy, intestinal metaplasia, and activity of gastritis.

### 2.5. Endoscopic Findings

Local anesthesia was the same as that for conventional gastroscopy. The gastroscopic procedures were performed using an upper GI video endoscope (Olympus EVIS EXERA III, CV-190). The whole stomach was examined first with conventional endoscopy. After the whole stomach mucosa was observed we chose site of specific gastric mucosa according to previous studies using magnification endoscopy [11–13]. The observed gastric mucosal morphologic pattern was classified into 5 morphologic patterns: type 1: regular arrangement of collecting venules, type 2: cone-shaped gastric pits, type 3: rod-shaped gastric pits with prominent sulci, type 4: ground glass-like pattern, and type 5: dark brown patches. Types 1 and 2 represent normal and mild inflammation morphologies, and types 3–5 represent moderate to severe inflammation morphologies (Figure 2) [9, 13].

### 2.6. Image Evaluation

All gastroscopic examinations were digitally recorded and still images of the observation sites were captured for the use in the reproducibility study. The selected images were transferred to a software program without distorting brightness, contrast, or color balance. All endoscopists classified them as type 1 through type 5 gastric mucosal morphologic patterns as described above. A total of 200 pictures from 200 patients were selected for the inter- and intraobserver agreement study. All endoscopists were blinded to the results of the* Helicobacter pylori* status and histology before reviewing the slides.

### 2.7. Statistical Analysis

The sensitivity, specificity, positive predictive value, and negative predictive values were calculated. Correlation with histopathological severity performs using Chi-square test. A *P* value of < 0.05 was considered significant. All statistical analyses were performed using the SPSS, version 16.0 (SPSS Inc., Chicago, IL, USA). The *κ* value was calculated for inter- and intraobserver variability. Interobserver variation was calculated from the results of the first reading, with 3 pairs in all. Intraobserver variation was determined by comparing the first and second assessment of each endoscopist, with 3 pairs in all. *κ* values below 0.4 indicated poor agreement, values between 0.4 and 0.6 represent moderate agreement, values between 0.6 and 0.8 represented substantial agreement, and values greater than 0.8 corresponded to excellent agreement.

## 3. Results

A total of 200 consecutive patients (92 men, 118 women; mean age: 49.0 years, range: 19–69 years) were enrolled in the study from January 2014 to November 2014. The 200 patients included 35 patients showing a type 1 pattern, 25 patients showing a type 2 pattern, 29 patients showing a type 3 pattern, 51 patients showing a type 4 pattern, and 60 patients showing a type 5 pattern (Table 2).* Helicobacter pylori* infection was demonstrated by both a positive result in the rapid urease test and the presence of bacteria seen on histological examination in 134 patients (67%).

**Figure 1:**
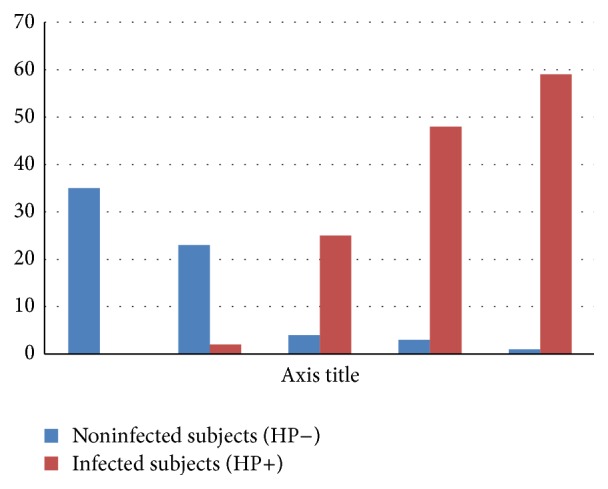
Correlation between gastric mucosal morphologic patterns and* Helicobacter pylori* infection status.

**Figure 2:**
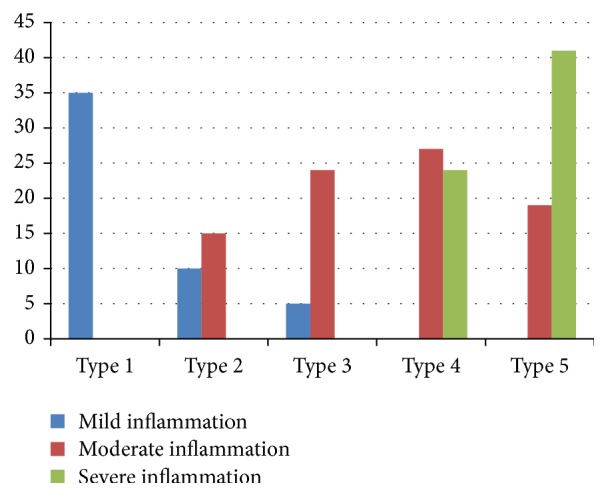
Correlation between gastric mucosal morphologic patterns and inflammation grading.

Type 1 and type 2 gastric mucosal morphologic patterns were statistically significant in predicting* Helicobacter pylori* negative status as compared with other mucosal morphologic patterns (58/60, *P* < 0.01) (Figure 1). Type 3, type 4, or type 5 gastric mucosal morphologic patterns were statistically significant in predicting* Helicobacter pylori* positive status as compared with other mucosal morphologic patterns (132/140, *P* < 0.01). Furthermore, the sensitivity, specificity, and positive and negative predictive values of type 3, type 4, or type 5 morphologic patterns for predicting the* Helicobacter pylori* positive gastric morphologic patterns were 94.28%, 96.66%, 98.50%, and 87.87%, respectively, with good correlation between gastric mucosal morphologic patterns and the severity of inflammation (*P* < 0.01).

### 3.1. Gastric Mucosal Morphology and Severity of Gastric Mucosal Inflammation

**Figure 3:**
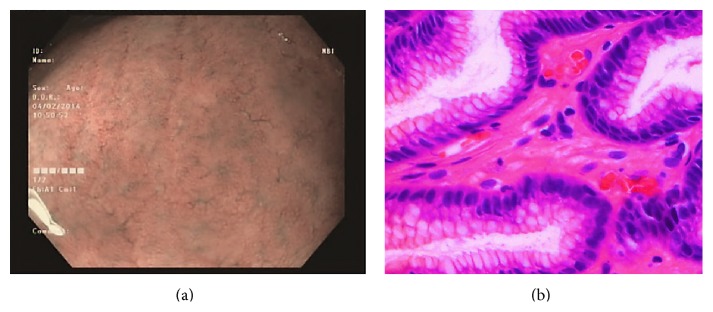
Type 1 regular arrangement of collecting venules (a). This pattern is associated with regular arrangement of surface epithelium, with absent infiltration by inflammatory cells (b).

**Figure 4 fig4:**
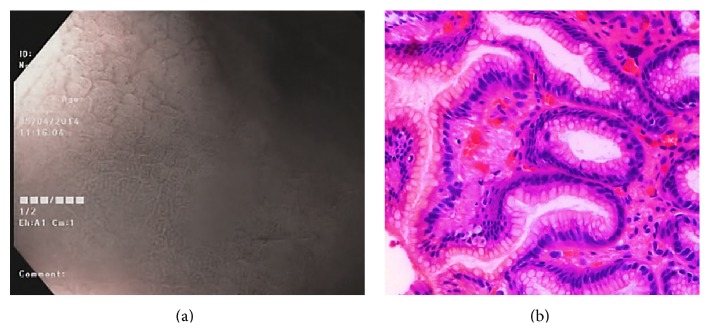
Type 2 cone-shaped gastric pits (a); abnormal C-NBI gastric mucosal morphologic patterns corresponded to mild gastritis with mild glandular atrophy, mild infiltration by inflammatory cells, irregular arrangement of surface epithelium, and irregular opening pits (b).

**Figure 5 fig5:**
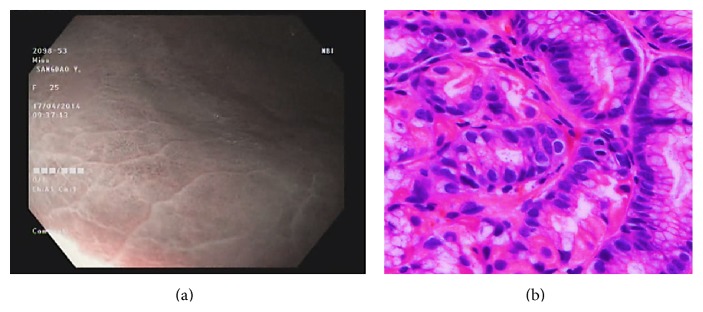
Type 3 rod-shape gastric pit with prominent sulcus (a). This pattern is associated with moderate glandular atrophy, moderate infiltration by inflammatory cells, and irregular arrangement of surface epithelium (b).

**Figure 6 fig6:**
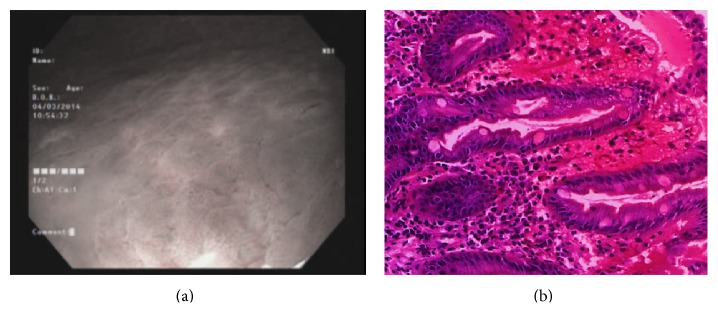
Type 4 ground glass-like patterns (a). Marked gastritis was found in type 4, with marked glandular atrophy, marked lymphocytic infiltration, lymphoid follicular hyperplasia, and mild intestinal metaplasia (b).

**Figure 7:**
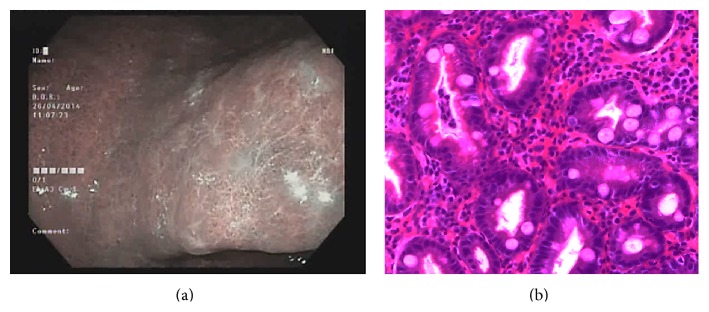
Type 5 brownish patches with bluish margin and irregular border (a). This pattern corresponded to marked intestinal metaplasia with marked gastric atrophy, indicating advanced gastritis (b).

Type 1 gastric mucosal morphologic patterns were associated with regular arrangement of surface epithelium, with no infiltration by inflammatory cells (Figure 3). Type 2 abnormal C-NBI mucosal morphologic patterns corresponded to mild gastritis with mild glandular atrophy, mild infiltration by inflammatory cells, irregular arrangement of surface epithelium, and irregular opening pits (Figure 4). Moderate gastritis was recognized in type 3, with moderate glandular atrophy, moderate infiltration by inflammatory cells, and irregular arrangement of surface epithelium (Figure 5). Marked gastritis was found in type 4, with marked glandular atrophy, marked lymphocytic infiltration, lymphoid follicular hyperplasia, and mild intestinal metaplasia (Figure 6). The dark brown patches in type 5 corresponded to marked intestinal metaplasia with severe gastric atrophy (Figure 7), indicating advanced gastritis.

### 3.2. Different C-NBI Gastric Mucosal Morphologies and Correlation with Histopathology

#### 3.2.1. Inter- and Intraobserver Agreement Assessment

The *k* values for inter- and intraobserver agreement for the gastroscopic mucosal morphologic patterns were significant. The *k* values for inter- and intraobserver agreement with regard to the prediction of* Helicobacter pylori* infection status were also significant (Table 4).

## 4. Discussion

Given that evidence from histopathology remains the gold standard for diagnosing* Helicobacter pylori* infection, the reliability of detecting* Helicobacter pylori* associated gastritis and related conditions such as atrophy and intestinal metaplasia by “*random biopsy*” sampling of gastric mucosa largely depends on the site, number, and size of biopsy specimens. Such a practice of random sampling can result in sampling errors, missed pathology, unnecessary work for pathology departments, and increase in costs of investigations. Early diagnosis and eradication of* Helicobacter pylori* infection are a key step in eliminating cancer risk. Real time identification of the area of* Helicobacter pylori* infection in the stomach during gastroscopy not only reduces the sampling error and excessive work load of laboratories but also improves detection efficacy of early gastric malignant lesions albeit this procedure needs more meticulous examination of the whole stomach [11]. The development of high resolution magnifying gastroscopy markedly overcame these problems [12, 14, 15], yet the use of magnified imaging for routine daily screening for* Helicobacter pylori* infection is impractical. It is not only costly but also less widely available, and it takes more time. In addition, it requires special patient preparation and need for experienced endoscopist. In our study, the gastric mucosal morphologic patterns were classified into 5 morphologic patterns using C-NBI gastroscopy. Type 1 and type 2 gastric mucosal morphologic patterns both were statistically significant in predicting* Helicobacter pylori* negative status compared to other gastric mucosal morphologic patterns (*P* < 0.01), whereas type 3, type 4, or type 5 gastric mucosal morphologic patterns were statistically significant in predicting* Helicobacter pylori* positive status as compared to the other gastric mucosal morphologic patterns (*P* < 0.01). These 3 gastric mucosal morphologic patterns (types 3, 4, and 5) were combined for analysis, yielding a good sensitivity, specificity, and positive and negative predictive values (94.28%, 96.66%, 98.50%, and 87.87%, resp.) for diagnosis of* Helicobacter pylori* infection. The Sydney system with the description and classification of histological severity of gastritis has become well established, whereas no international consensus has yet been reached on the description and classification of specific gastroscopic gastritis findings. The present study of the correlation between gastric mucosal morphologic pattern and histological gastritis severity (using the updated Sydney classification) shows a good correlation between the gastric mucosal morphologic pattern and the severity of gastritis (*P* < 0.01).

In conclusion, considering the unsatisfactory sensitivity of conventional gastroscopy for diagnosing* Helicobacter pylori* associated gastritis and the limitations of magnified endoscopic imaging technique, our study suggests that gastric mucosal morphologic pattern in* Helicobacter pylori* infected gastric mucosa can be reliably identified using C-NBI gastroscopy and can also predict the histopathological severity of gastritis.

### 4.1. Comment and Future Direction

**Figure 8:**
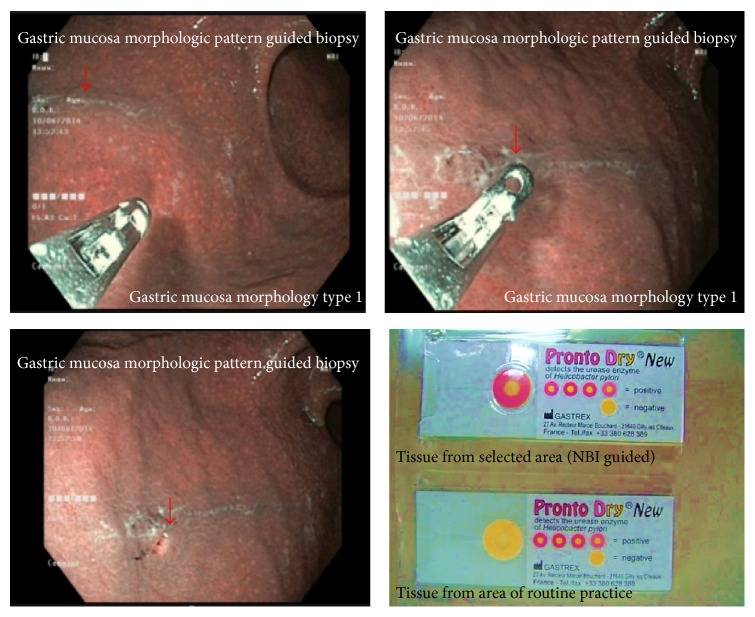
Site specific biopsy.

Our study found good correlation between gastric mucosal morphologic pattern,* Helicobacter pylori* status, and severity of pathological inflammation grading (Table 3). Thus gastric mucosal morphologic pattern may be useful for “*site specific biopsy*” of the areas with suspected* Helicobacter pylori* infection in daily practice (Figure 8). We plan to compare the gold standard biopsy technique with site specific biopsy technique in the next study.

## Figures and Tables

**Table 1:** Updated Sydney system.

Histologic properties	Definition	Grade		
		Mild	Moderate	Severe
Chronic inflammation	Lymphocyte and plasma cell in lamina propria	1+	2+	3+
Neutrophil activation	Neutrophilic infiltration in lamina propria or superficial epithelium	<1/3	1/3–2/3	>2/3
Glandular atrophy	Loss of corpus and antral glands	1+	2+	3+
Intestinal metaplasia	Intestinal metaplasia of mucosal epithelium	<1/3	1/3–2/3	>2/3
*Helicobacter pylori *	*Helicobacter pylori* intensity	1+	2+	3+

**Table 2:** Correlation between gastric mucosal morphologic patterns and *Helicobacter pylori* infection status.

Mucosal morphology	*Helicobacter pylori* infection status		*P*
	Noninfected subjects (HP−)	Infected subjects (HP+)	
Type 1	35 (35/35)^*^	—	<0.01
Type 2	23 (23/25)^*^	2 (2/25)	<0.01
Type 3	4 (4/29)	25 (25/29)^*^	<0.01
Type 4	3 (3/51)	48 (48/51)^*^	<0.01
Type 5	1 (1/60)	59 (59/60)^*^	<0.01

^*^Statistical significant.

**Table 3:** Correlation between gastric mucosal morphologic patterns and inflammation grading.

Mucosal morphology	Inflammation grading			*P*
	Mild	Moderate	Severe	
Type 1	35 (35/35)	—	—	<0.01
Type 2	10 (10/25)	15 (15/25)	—	<0.01
Type 3	5 (5/29)	24 (24/29)	—	<0.01
Type 4	—	27 (27/51)	24 (24/51)	<0.01
Type 5	—	19 (19/60)	41 (41/60)	<0.01

**Table 4:** Inter- and intraobserver agreement.

	Interobserver agreement		Intraobserver agreement	
	% agreement	*k* value (95% CI)	% agreement	*k* value (95% CI)
Gastric mucosal morphology	98.8	0.98 (0.97–0.98)	89.9	0.89 (0.87–0.89)
*H. pylori *infection status	97.9	0.94 (0.92–0.95)	98.3	0.97 (0.96–0.97)
